# Mycotic pseudoaneurysm of the extracranial carotid artery, a severe and rare disease, a case report

**DOI:** 10.1016/j.ijscr.2020.03.032

**Published:** 2020-04-01

**Authors:** Gabriel Molina, Carolina Mesías, Juan Calispa, Kevin Arroyo, Katherine Jaramillo, Ligia Lluglla, Bernardo Gutierrez, Patricio Gálvez

**Affiliations:** a PGY4 Surgery Resident at Pontificia Universidad del Ecuador, Quito, Ecuador; b Head and Neck Surgery, Universidad Autónoma de México, Mexico; c Hospital de Especialidades Fuerzas Armadas, Quito, Ecuador; d Universidad Central del Ecuador, Department of Surgery, Quito, Ecuador; e Pontificia Universidad Católica del Ecuador, Department of Surgery, Quito, Ecuador; f Universidad Central del Ecuador, Quito, Ecuador; g Universidad San Francisco, Quito, Ecuador; h Docente Carrera de Medicina, Facultad de Ciencias Médicas, Universidad de las Américas, Quito, Ecuador

**Keywords:** Mycotic pseudoaneurysm, Carotid artery, Neck surgery

## Abstract

**Background:**

Mycotic pseudoaneurysm of the carotid artery is a severe and rare disease. When it ruptures, it can produce severe morbidity and high mortality. It presents clinically as a pulsatile mass, usually diagnosable by computed tomography angiography with definitive confirmation achieved by establishing the presence of the pathogen in the tissue sample.

**Case presentation:**

We present the case of a 68-year-old male patient with a history of total laryngectomy. He presented with painful and pulsatile mass in his neck and, after precise evaluation, a ruptured carotid mycotic pseudoaneurysm was promptly detected and treated. After a surgical intervention and an adequate course of antibiotics, the patient successfully recovered.

**Conclusions:**

Mycotic pseudoaneurysms of the extracranial carotid artery are uncommon and should always be surgically treated. Due to the high risk of potential complications, restoration of the arterial flow should be attempted in all occasions. High clinical awareness is imperative when approaching a mycotic pseudoaneurysm due to its wide spectrum of clinical symptoms, and must always be considered when diagnosing tumors of the neck. Despite its rarity, early detection and prompt treatment are critical to minimize the possibility of a fatal outcome.

## Highlights

• Mycotic pseudoaneurysm of the carotid artery is a severe and rare disease.
• High clinical awareness is imperative when approaching a mycotic pseudoaneurysm due to its wide spectrum of clinical symptoms, and must always be considered when diagnosing tumors of the neck.
• Early detection and prompt treatment are critical as this disease though rare can have a fatal outcome.

## 1 Introduction

### 1.1 This work has been reported in line with the SCARE criteria

Ruptured carotid mycotic pseudoaneurysms are a rare yet severe condition. Generally speaking, mycotic pseudoaneurysms are localized, irreversible arterial destruction of the vessel by an infection. The term was described by Osler in 1885, and is employed to highlight false aneurysms that appear on a vessel associated to an infection. Clinically, they present as a growing, pulsatile cervical mass associated with pain, tenderness, fever, dysphonia, and dysphagia [1,2]. Preoperative diagnosis is challenging due to the plethora of unspecific symptoms that characterize the condition, particularly when no known risk factors are reported [3]. Upon suspicion, the use of computed tomography angiography (CTA) at the preoperative stage is vital, as it gives detailed anatomy and outline surgery. Beyond a structural characterization, definitive diagnosis is achieved by identifying the presence of a microbial pathogen in the tissue sample [2], which is usually bacterial despite the name of the condition [3]. Surgical removal is the treatment of choice, along with the reconstruction of the damaged artery [1,4]. Following these lines, we present the case of a male patient with a pulsatile mass on his neck, successfully diagnosed as a carotid pseudoaneurysm. The mass was successfully surgically removed, and the patient fully recovered.

This work has been reported in line with the SCARE criteria [12].

## 2 Case report

The patient is a 68-year-old male with a past medical history of larynx carcinoma and a radical laryngectomy with bilateral neck dissection. The surgery was performed more than 6 years ago and he never presented complications attributable to the intervention. He arrived at the emergency room (ER) with a high fever and a painful left neck mass. 15 days before arriving at the ER, he developed a general feeling of unwellness, along with high fever and the aforementioned pulsating mass in the neck which had been increasing in size ever since. During the previous 24 h, he developed severe pain around the mass and progressive shortness of breath. There was no history of neck trauma, infection or stroke.

**Fig. 1:**
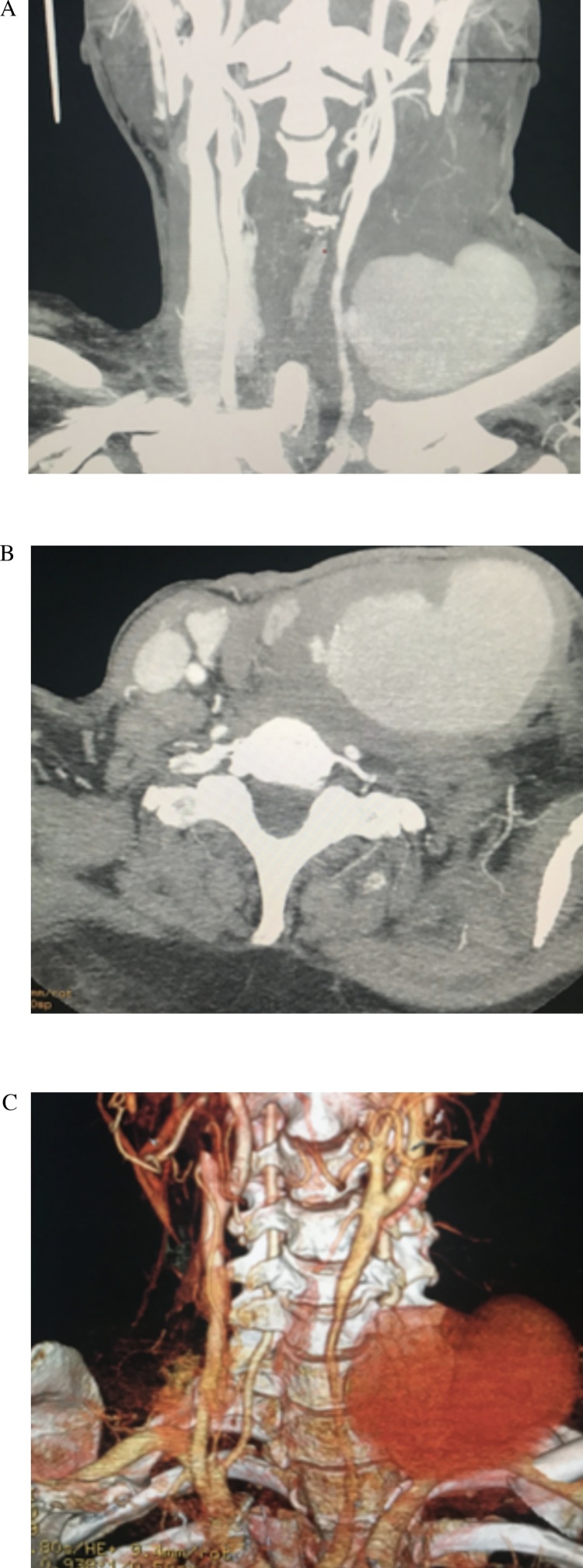
A: Neck mass near the left common carotid artery. B: Mycotic pseudoaneurysm on the left common carotid artery. C: 3D Reconstruction of the mycotic pseudoaneurysm.

On physical examination, a tachycardic, febrile, and dehydrated patient was encountered. Along with a 15 × 5 × 2 cm beating and painful mass in the left region of his neck, general swelling was observed and the patient reported severe pain on touch. Also, a carotid murmur was audible on auscultation. The rest of his physical examination was unremarkable. After adequate resuscitation, complementary exams revealed leukocytosis with neutrophilia, along with anaemia. Due to the size of the mass, and to aid in the preoperative planning, a computed tomography angiography (CTA) was requested. The results revealed a 10 × 5 × 2 cm hyper-enhanced lobulated lesion involving the left common carotid artery (CCA) and carotid bulb (Fig. 1A, B), and severe swelling of the neck tissues (Fig. 1C). A diagnosis of pseudoaneurysm of the left CCA was suspected, requiring a vascular surgery consultation; an emergency surgery was decided upon deliberation.

**Fig. 2:**
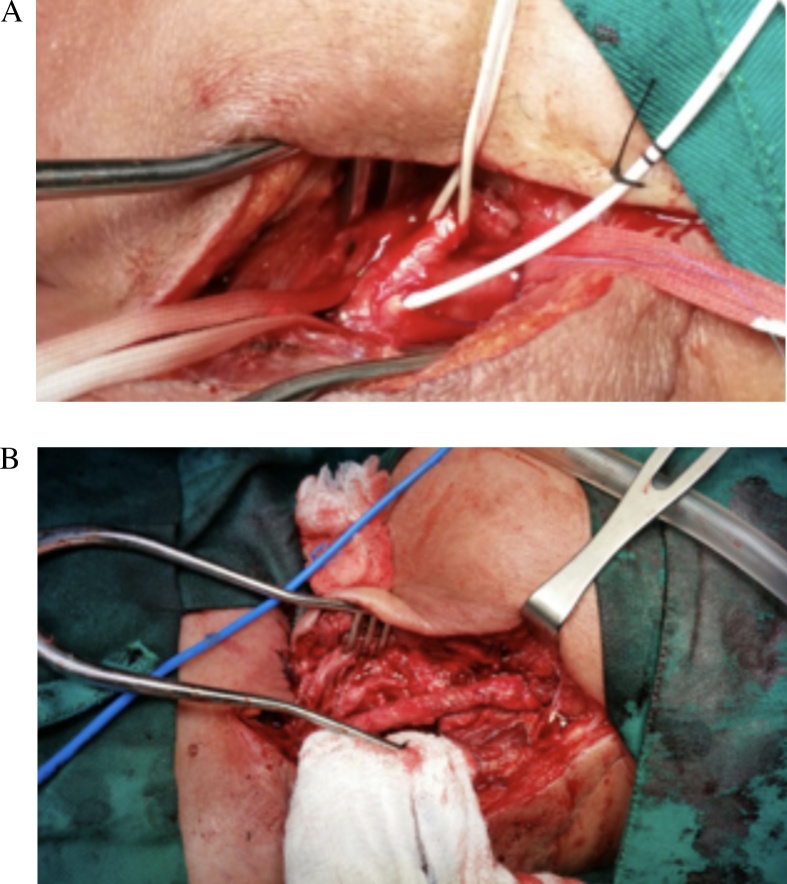
A: Aneurysmal sac during surgery. B: Saphenous vein graft bypass after debridement.

Broad-spectrum antibiotics were initiated and a left neck cervicotomy was achieved with exposure of the left common carotid artery. The circumference of the mass was dissected, and the proximal and distal CCA regions were identified and clamped. The aneurysmal sac was opened and we discovered a ruptured wall of the CCA with inflammation, necrosis and 150 cc of pus. After exhaustive washing of the wound, and since the left carotid artery had some ischemic patches, a saphenous vein graft bypass (Fig. 2A) was used to reconstruct the damaged artery (Fig. 2B).

The postoperative course was unremarkable. A histologic examination of the arterial wall identified the presence of a mycotic pseudoaneurysm with evidence of organisms (*Staphylococcus aureus*). The bacteriologic profile of the tissue sample showed that the identified *S. aureus* presented sensitivity to the vancomycin, and therefore the patient received a 10-day cycle of intravenous vancomycin. He was discharged without any complications and, on follow-up controls up to one year after surgery, the patient is doing well.

## 3 Discussion

Mycotic pseudoaneurysms fall within one of two categories: actual mycotic pseudoaneurysms arise following infection of a previously normal arterial wall, whereas an infected traumatic pseudoaneurysm is an infection of a pre-existing aneurysm [3,14]. In this case, our patient didn't have a history of a pre-existing aneurysm, neck trauma or drug abuse corroborated by the series of close follow-up controls due to his previous carcinoma. In fact, the most common cause of mycotic pseudoaneurysms is trauma (42%), but in 25% of the cases the exact source of infection is unknown [13]. Mycotic pseudoaneurysms of the carotid artery are rare events, and account for only 5% of cases of pseudoaneurysms [1,2]. They are formed due to chronic inflammation and hemorrhage in the setting of an infectious process that affects the arterial wall. They can appear due to a primary infection and lead to perforation and an abscess, although some authors suggest that the abscess itself is the primary lesion that perforates into the artery. Other local vessel inflammation disorders such as vasculitis or hereditary dysfunctions could weaken the arterial wall and predispose it to infection [1,4]. In our 68-year-old patient, his previous neck surgery may have contributed in some way to the development of the mycotic pseudoaneurysm, but there was no clear evidence for this and the exact cause of the infection could not be established

The nonspecific symptomatology of mycotic pseudoaneurysms includes cervical pain, tenderness, dysphonia, dysphagia, and a growing and pulsatile cervical mass [5]. Auscultation can sometimes reveal the presence of a murmur or thrill, and a purplish or brownish discoloration of the skin has been described in some cases [6]; both symptoms were found on our patient. Diagnosis is achieved through a description of the clinical symptomatology and a combination of CT, angiography and/or ultrasound. Definitive diagnosis is ultimately based on the presence of the bacteria in the arterial wall, with the most common discovered pathogens being *Staphylococcus aureus*, *Salmonella* spp. and different Streptococci [7] (as it was discovered on our patient).

Management of mycotic pseudoaneurysms includes the administration of systemic antibiotics and surgical resection. Vascular reconstruction due to the high risk of thrombosis, embolization, and rupture is also key, as these complications have been reported in up to 10% of the cases [5,8]. Surgical management includes excision of the pseudoaneurysm and restoration of the arterial continuity with debridement of the infected tissues. Vascular reconstruction is achieved using a variety of techniques including primary closure, patch angioplasty, bypass, and resection with primary anastomosis. Autologous arteries, veins, synthetic prosthesis impregnated with silver salts, or cryopreserved arterial allografts have all been used, but the risk of secondary deterioration from stenosis or dilatation related to the infectious processes is still a concern. The superficial femoral artery, the hypogastric artery and the saphenous vein have all been used as well, and the latter is currently considered the preferred material for vascular reconstruction because of its resistance to infection and its immediate availability [1,2,16]. Endovascular repair with covered stents or coils is an interesting alternative for embolization of carotid mycotic pseudoaneurysms, but its utility is limited by the risks associated with introducing prosthetic material into an infected area. Nevertheless, the use of a covered stent, in combination with long-term antibiotic therapy as a bridge to the classical surgical intervention has been successful in numerous stable patients [1,2,6,13,15].

A particular emphasis should be placed on the ligation of the CCA. This procedure should be performed only when there is evidence of an intact Circle of Willis, as this intervention carries a mortality risk of 25%–60% and a high incidence of stroke [6,9]. Endovascular procedures can be attempted on certain occasions, bearing in mind that the stent placement may cause thrombus at the time of the procedure (therefore requiring anticoagulation) or could become deformed, migrate, or occlude the vessel [10,11]. The course of action chosen for our patient after the detection of the mycotic pseudoaneurysm through an angiography was completed without complications.

Mycotic pseudoaneurysms of the extracranial carotid artery are uncommon and should always be surgically treated, with an emphasis on the restoration of the arterial flow due to the high risk of complications [1,2,8]. Due to its rarity and wide spectrum of clinical symptoms, high clinical awareness is imperative when approaching a suspected mycotic pseudoaneurysm, and when diagnosing tumors of the neck. Early detection and prompt treatment are critical as this disease, though rare, can have a fatal outcome.

## Declaration of Competing Interest

The authors declares that there is no conflict of interest regarding the publication of this article.

## Funding

The authors have no funding to report.

## Ethical approval

The authors declare that = we obtained permission from the ethics committee in our institution.

## Consent

The authors declare that written consent was obtained from the patient before publication of this case.

## Registration of research studies

The authors declare that the patient gave his consent to publish this case, and as this is a case report not human participants were involved in a study.

## Guarantor

Patricio Gálvez MD, Docente Carrera de Medicina, facultad de ciencias médicas, Universidad de las Américas, Quito-Ecuador.

## Provenance and peer review

Not commissioned, externally peer-reviewed.

## CRediT authorship contribution statement

**Gabriel Molina:** Conceptualization. **Carolina Mesías:** Conceptualization, Data curation, Formal analysis. **Juan Calispa:** Data curation. **Kevin Arroyo:** Data curation. **Katherine Jaramillo:** Data curation. **Ligia Lluglla:** Conceptualization, Data curation, Formal analysis. **Bernardo Gutierrez:** Data curation. **Patricio Gálvez:** Conceptualization, Data curation, Formal analysis.
